# The Healing Power of Clean Rivers: In Silico Evaluation of the Antipsoriatic Potential of Apiin and Hyperoside Plant Metabolites Contained in River Waters

**DOI:** 10.3390/ijerph19052502

**Published:** 2022-02-22

**Authors:** Valentina Roviello, Melinda Gilhen-Baker, Caterina Vicidomini, Giovanni N. Roviello

**Affiliations:** 1 Department of Chemical, Materials and Industrial Production Engineering (DICMaPI), University of Naples Federico II, Piazzale V. Tecchio 80, 80125 Naples, Italy; valentina.roviello@unina.it; 2 Faculty of Physical Medicine and Rehabilitation, Georgian State Teaching University of Physical Education and Sport, 49, Chavchavadze Avenue, 0162 Tbilisi, Georgia; mgbaker@me.com; 3 Istituto di Biostrutture e Bioimmagini IBB—CNR, Area di Ricerca site and Headquartes - Via Pietro Castellino 111, 80131 Naples, Italy; caterina.vicidomini@ibb.cnr.it

**Keywords:** environmental health, plant metabolites, phytomedicine, anti-psoriatic drug, molecular docking

## Abstract

Humanity may benefit greatly from intact riverine ecosystems not only because they supply water to be used in the most common human activities, but also for the effects that clean rivers can have on human health. Herein, we used a computational approach to show that some phytochemicals produced by riparian plants as secondary metabolites, which are naturally released into river waters, can have therapeutic properties. These include antipsoriatic activities which we demonstrated in silico by modelling the interaction of apiin, guanosine and hyperoside, a few main river plant metabolites, with NF-kB, IL-17 and IL-36, which are recognized targets involved in psoriasis disease. In particular, we found that apiin and hyperoside are endowed with docking energies and binding affinities which are more favorable than the known reference inhibitors of the three protein targets whilst, in silico, guanosine shows comparable activity with respect to the inhibitors of IL-36 and NF-kB. The low skin permeation (logKp < −8) we predicted for apiin and hyperoside led us to hypothesize their possible utilization as topic antipsoriatic therapeutics, and in particular after PAINS (pan-assay interference compounds) score evaluation, we reached the conclusion that apiin, with no predicted tendency to react nonspecifically with the numerous targets involved in the biological cellular pathways, is particularly interesting for the desired therapeutic application.

## 1. Introduction

**Figure 1:**
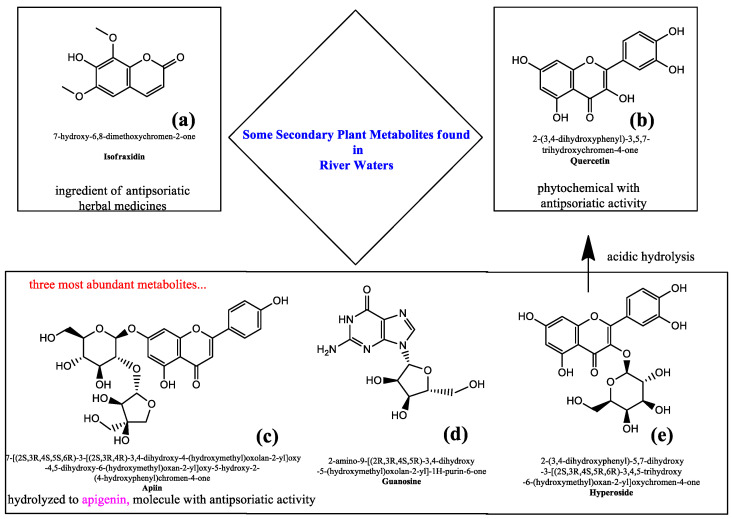
Chemical structures of some of the plant metabolites (**a**–**e**) detected in river waters. Note how the most abundant apiin (**c**), guanosine (**d**) and hyperoside (**e**) subjected to the in silico analysis of the current work are shown in the rectangular box, while isofraxidin (**a**), contained in an antipsoriatic treatment, and quercetin (**b**), with known antipsoriatic activity and in turn obtained after acidic hydrolysis of hyperoside, are represented in the upper part of the figure.

Since antiquity, rivers have served as an important source of food and water as well as a natural waterway for transportation and navigation. Like many important natural features, they have also been considered sacred to cultures around the world for their life giving qualities and have been compared to arteries in the human body. The Ganges in India, the Nile in Egypt and the Amazon River are but a few famous waterways that have been and continue to be central to the survival and culture of the surrounding peoples [1]. The immense importance placed on these water ways for their wide range of benefits and uses has pushed countries such as India and New Zealand to bestow on the Ganges and the Whangananui Rivers the legal status of persons and the consequent legal rights in order to better protect them [2]. Rivers of course also play a central role in the ecology of the planet as vibrant and vital ecosystems, which house numerous species [3]. Nowadays, the long list of common uses of rivers also includes recreational activities such as bathing, walking, camping, and fishing [4,5]. River water cleanliness and consequent suitability for swimming and ecosystem biodiversity, including abundance of fish species or pollution-sensitive river mammals [6], are key indicators of good aquatic health. These guiding points influence whether or not potential users decide to make use of rivers for recreational purposes, which include those that do not necessarily involve any contact with the river water such as fishing, walking, and engaging in other sporting activities near the river [4]. Sadly, the impairment of biodiversity and river health are causing a decrease in recreational use of rivers, [4] making the use of these waterways, and their therapeutic benefits, much less common. Among the most common disruptions endured by river systems, very evident are the alterations of the riverine habitat structure due to human intervention and the consequent loss of harmony within the fluvial landscape [4]. This can be due to dams and bridges being built, as well shoreline destruction for naval or recreational purposes. Numerous are the consequences of such disturbances which are compounded by the harm caused by the chemical pollution of fluvial ecosystems such as faecal water contamination and eutrophication in urban areas [7,8], migration of pollutants from heating plants to rivers [9], microplastics [10], herbicide water contamination [11], river sediment pollution by various harmful hydrocarbons [12] and chloride contamination from road salt application in colder climates [13] to mention just a few. Lower recreational use of rivers is mainly associated with diminished water transparency following algal and bacterial blooms which create an immediate negative visual perception of the river’s safety [4]. Air pollution in the vicinity of polluted rivers, from which anthropogenic volatile organic compounds can be released forming photochemical smog, may also heavily affect the health of residents living along the riverside [14,15]. Therefore, the protective effects of biogenic volatile organic compounds at the basis of the emerging ‘green prescriptions’ and ‘forest-bathing’ [16,17,18,19] that could derive in principle from riparian plant emission are outweighed by the adverse effects of anthropogenic volatile organic compounds. Herein, we aim at discussing the potential benefits provided by clean rivers on human health from a therapeutic perspective using in silico techniques to show that some secondary plant metabolites which are relatively abundant in river waters could provide benefits in the treatment of psoriasis. To this aim, conscious of the role of molecular docking (MD) in the exploration of biomedically-relevant molecular interactions, we conducted an MD study with the program HDOCK [20,21,22,23,24,25] using the 3D structures of NF-kB, IL-17 and IL-36, that are the protein targets involved in psoriasis [26] and those from three plant metabolites found in rivers. HDOCK is used for both macromolecule-macromolecule [20,21,22,23] and small molecule-macromolecule [24] dockings, with types of macromolecules including proteins. HDOCK score furnished by the program is an energy score whose values are listed dimensionless [25] and larger negative numbers of HDOCK score indicate stronger binding interactions between the interacting ligand/macromolecules, which was reported to correlate well to experimental binding affinities [25]. Different flavonoids and their glycosilated forms (Figure 1) were identified by liquid chromatography coupled to high resolution mass spectrometry (LC-HRMS) as secondary plant metabolites released into river waters from riparian vegetation at concentrations up to about 5 µg/L [27]. These river plant metabolites, produced in great variety depending on the plant species, season and environmental conditions, play a key role in controlling essential functions such as plant growth and reproduction, and in establishing bioecological relationships with other organisms [27]. A recent study conducted on German floodplain forests along the rivers Elster, Pleiße and Luppe showed that among the plethora of secondary metabolites from riparian vegetation, apiin (produced from Apiaceae and Urtica dioica), guanosine (from Digitalis purpurea), and hyperoside (from Hypericum species, Fraxinus excelsior and Galanthus nivalis) were those present at the highest concentrations (up to about 5 µg/L) [27] within the other compounds detected in the waters of the rivers. Ultimately, our work emphasizes the need to preserve the quality of river water in order to entice people back to the once popular habit of frequenting rivers for recreational purposes, especially in the warmer seasons, which could lead to positive effects on human health.

## 2. Materials and Methods

### 2.1. Molecular Docking

In the molecular dockings, we used the HDOCK server [20,21,22,23,24] (with default parameters) to which we furnished as 3D structures of NF-kB, IL-17 and IL-36 -the protein targets involved in psoriasis subjects of our investigation – those with (Protein Data Bank [PDB] ID: 1A3Q, 4HSA, and 6P9E, respectively. HDOCK server uses the iterative knowledge-based scoring function ITScore-PP to rank the top 10 poses provided after each docking run [23]. The structures for the natural compounds and the literature inhibitors of the three proteins were retrieved by us from the PubChem database (https://pubchem.ncbi.nlm.nih.gov/, accessed on 8 November 2021) and corresponded to the entries PubChem CID 5280746 (apiin), 5281643 (hyperoside), and 135398635 (guanosine). More details on HDOCK docking server and on the procedures for docking experiments can be found at http://hdock.phys.hust.edu.cn/ (accessed on 9 November 2021). The docking method was validated using as reference compounds the known inhibitors [26]:

- (6aS,10aS)-9-(hydroxymethyl)-6,6-dimethyl-3-(2-methyloctan-2-yl)-6a,7,10,10a-tetrahydrobenzo[c]chromen-1-ol (dexanabinol, NF-kB);
- (4S,20R)-7-chloro-N-methyl-4-{[(1-methyl-1H-pyrazol-5-yl)carbonyl]amino}-3,18-dioxo-2,19-diazatetracyclo[20.2.2.1_6,10_.1_11,15_]octacosa-1(24),6(28),7,9,11(27),12,14,22,25-nonaene-20-carboxamide (IL-17);
- (2S)-2-{[4-(3-amino-4-methylphenyl)-6-methylpyrimidin2-yl]oxy}-3-methoxy-3,3-diphenylpropanoic acid (IL-36).

In our blind docking, the reference compounds bound the respective protein targets involving amino acid residues (Table 1, Table 2 and Table 3) that were reported in the literature as binding residues for the given complexes [26]; moreover, we analyzed the top-ranked poses (Top-1) and the top 10 ranked poses for the complexes predicted by HDOCK according to the energy scores provided by the program as explained in the Results and Discussion section. Ligand/Protein complexes were visualized in the structure viewer of the HDOCK server.

### 2.2. Prediction of Chemico-Physical and Pharmacokinetic Properties

LogS and LogKp (skin permeation: the more negative the log Kp, the less skin permeant is the molecule) values were predicted by SwissADME software (http://www.swissadme.ch/index.php accessed on 8 November 2021), together with chemico-physical properties and pan-assay interference compounds (PAINS) scores.

All these properties shown in Tables S1 and S2 were computed using the isomeric SMILES (Simplified Molecular Input Line Entry System) format for the compounds found in the PubChem database:

SMILES:

C1[C@@]([C@H]([C@@H](O1)O[C@@H]2[C@H]([C@@H]([C@H](O[C@H]2OC3=CC(=C4C(=C3)OC(=CC4=O)C5=CC=C(C=C5)O)O)CO)O)O)O)(CO)O (apiin)

SMILES:

C1=CC(=C(C=C1C2=C(C(=O)C3=C(C=C(C=C3O2)O)O)O[C@H]4[C@@H]([C@H]([C@H]([C@H](O4)CO)O)O)O)O)O (hyperoside)

## 3. Results and Discussion

Psoriasis is a chronic autoimmune skin disease affecting more than 2% of the population around the globe, characterized by keratinocyte hyperplasia, with inflammatory cell infiltration into the dermis and neovascularization [28]. The main psoriasis protein targets in the antipsoriatic drug discovery are NF-κB, IL-17, and IL-36 which can be subjected to molecular docking investigations for screening potential drug candidates [26]. Antipsoriatic treatments are typically searched in several classes of compounds including plant-derived products such as diterpenoids, triterpenoids [26], and hydroxycinnamic acids [29]. 

Interestingly, among the secondary plant metabolites detected in river waters and shown in Figure 1, isofraxidin (Figure 1a) is contained in an antipsoriatic herbal product which was found able to inhibit keratinocyte proliferation downregulating the cyclin B2 [28]. Additionally, the river plant metabolite quercetin (Figure 1b), the hydrolysis product of hyperoside (Figure 1e), exerted in vitro and in vivo anti-inflammatory effects resulting in promising antipsoriatic agents [30].

**Table 1:** Ligand/NF-kB complexation: HDOCK docking scores (for the top ranked poses and averaged on the Top-1–10 poses). The main amino acids involved in the ligand/protein complexes for the Reference compound and each plant metabolite are reported in the last column.

	HDOCK Score ^a^ for the Top-1 Ranked Pose	HDOCK Score ^a^—Averaged on the Top 10 Poses ± SD	Main Residues Involved in the Ligand-Protein Complex
Reference compound ^b^	−253.91	−210.047 ± 25.277	Tyr55, Ser220, Asn227, Lys252, Gln284
Apiin	−311.83	−275.535 ± 24.457	Gly50, Ser220, Asn227, Lys252, Gln284
Guanosine	−210.04	−181.209 ± 16.489	Ser220, Lys252, Gln284
Hyperoside	−310.81	−286.252 ± 10.385	Ser220, Asn227, Lys252, Gln284

^a^ The docking energy scores. ^b^ Dexanabinol [26].

**Table 2:** Ligand/IL-17 complexation: HDOCK docking scores (for the top ranked poses and averaged on the Top-1–10 poses). The main amino acids involved in the ligand/protein complexes for the Reference compound and each plant metabolite are reported in the last column.

	HDOCK Score ^a^ for the Top-1 Ranked Pose	HDOCK Score ^a^—Averaged on the Top 10 Poses ± SD	Main Residues Involved in the Ligand-Protein Complex
Reference compound ^b^	−234.41	−213.715 ± 10.023	Asn36, Pro37, Leu97
Apiin	−249.73	−235.954 ± 9.371	Met166,Ser167, Trp193, Asn194
Guanosine	−183.72	−162.772 ± 10.651	Asp58, His131, Lys135, Pro136
Hyperoside	−248.10	−224.287 ± 10.313	Met166, Ser167, Trp193, Asn194, Tyr62, Pro63, Val65

^a^ The docking energy scores. ^b^ (4S,20R)-7-chloro-N-methyl-4-{[(1-methyl-1H-pyrazol-5-yl)carbonyl]amino}-3,18-dioxo-2,19-diazatetracyclo[20.2.2.1_6,10_.1_11,15_]octacosa-1(24),6(28),7,9,11(27),12,14,22,25-nonaene-20-carboxamide.

**Table 3:** Ligand/IL-36 complexation: HDOCK docking scores (for the top ranked poses and averaged on the Top-1–10 poses). The main amino acids involved in the ligand/protein complexes for the Reference compound and each plant metabolite are reported in the last column.

	HDOCK Score ^a^ for the Top-1 Ranked Pose	HDOCK Score ^a^—Averaged on the Top 10 Poses ± SD	Main Residues Involved in the Ligand-Protein Complex
Reference compound ^b^	−137.72	−125.561 ± 5.461	Arg121, Lys123, Val58, Leu165, Ile27
Apiin	−171.43	−156.13 ± 6.49	Arg121, Lys123, Leu165, Asn166
Guanosine	−115.74	−112.396 ± 3.126	Lys123, Leu165, Asn166
Hyperoside	−145.44	−140.679 ± 2.929	Arg121, Lys123, Leu165, Leu151

^a^ The docking energy scores. ^b^ (2S)-2-{[4-(3-amino-4-methylphenyl)-6-methylpyrimidin-2-yl]oxy}-3-methoxy-3,3-diphenylpropanoic acid.

The importance of phytochemicals in the antipsoriatic drug discovery prompted us to investigate whether the three main plant metabolites found in river water could be potentially useful in the treatment of this autoimmune disease. To this aim we made use of in silico methods, and more specifically of molecular docking in analogy to other recent literature examples using NF-kB, IL-17 and IL-36 as targets of tested drug candidates [26,29]. We found by HDOCK docking that the secondary plant metabolites apiin (Figure 1c) and hyperoside (Figure 1e) bound the targets with predicted binding energies lower (Table 1, Table 2 and Table 3) and, thus, affinities higher than the three respective reference inhibitors. 

**Figure 2:**
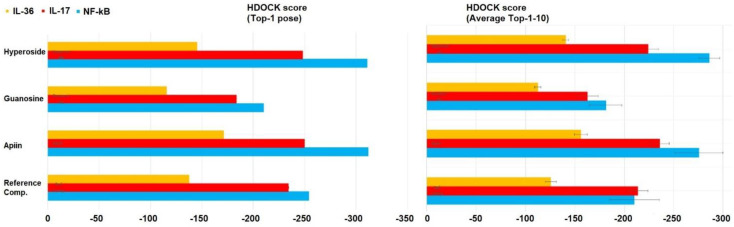
HDOCK scoring of complexes of the three protein targets (NF-kB, IL-17 and IL-36) involved in psoriasis investigated in our study with the plant metabolites hyperoside, guanosine, apiin and their respective reference inhibitors. Note how the scores for the top-ranked (Top-1) poses are presented in the left side of the figure, while the means (considering the values for poses Top1–10) with the respective Standard Deviations are shown on the right.

**Figure 3:**
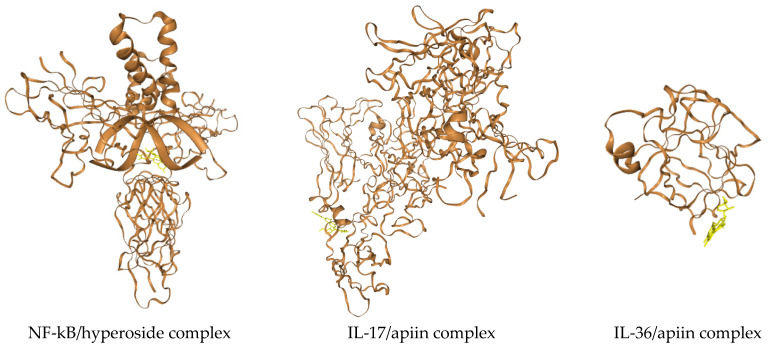
Structure representations of the complexes NF-kB/hyperoside (**left**), IL-17/apiin (**middle**), and IL-36/apiin (**right**) obtained by us after molecular docking. Note how plant metabolites are represented in yellow.

This emerged from the analysis of both the top ranked poses resulting from the molecular docking and the averaged score based on the top 10 poses (Figure 2). Guanosine (Figure 1d) showed comparable affinity with respect to the reference compound only in the case of IL-36 and NF-kB (considering the averaged score), but both guanosine and the reference compound were less effective binders of the same targets than apiin and hyperoside (Figure 2). These latter were endowed with similar binding affinities for all targets but IL-36 with which apiin showed slightly higher affinity (Figure 2) and whose complex structure is reported in Figure 3 next to those relative to the complexes of the other two proteins with hyperoside and apiin (NF-kB/hyperoside, IL-17/apiin, Figure 3). Interestingly, binding residues involved in case of IL-17 for the secondary plant metabolites were different than those found for the reference compound (Table 2), thus suggesting that binding involves a different protein region with apiin, guanosine and hyperoside with respect to the literature inhibitor of IL-17 [26]. In more detail, four of the main amino acids involved in the molecular interaction between apiin and hyperoside with NF-kB were also found for the reference compound with the same target (Ser220, Asn227, Lys252, Gln284), while in the case of guanosine the residues in common were only three (Ser220, Lys252, Gln284, Table 1). As for the complexes with IL-17, the phytochemicals of our study involved amino acid residues that differed from those found for the reference compound (Table 2). Owing to the ligands/IL-36 bindings, Arg121, Lys123, Leu165 were residues involved in the molecular recognition of both apiin and hyperoside with IL-36, while only the last two were found in the complex of guanosine with the same protein together with Asn166 (Table 3). Moreover, we predicted the chemico-physical and pharmacokinetic properties of the two promising antipsoriatic candidates emerged by the molecular docking by the SwissADME software and found similar solubilities (with LogS values of about −3) and low skin permeations (LogKp values lower than −8 cm/s) in both apiin and hyperoside (Tables S1 and S2). The only difference which emerged in this prediction consisted of the PAINS score that was found null only in the case of apiin (Table 1), for which we can exclude any tendency to interact nonspecifically with the numerous cellular biological targets. The importance of apiin as antipsoriatic is also corroborated by the finding that its hydrolysis product, apigenin, was able to alleviate psoriasis and improve the physical and chemical skin barrier functions [31]. The negative LogKp values suggest that apiin and hyperoside cannot penetrate the skin. On the other hand, a limited skin permeation is typically associated with drugs to be used topically without any possible toxicity side effects through skin contact [32]. In this regard, many antipsoriatic treatments are based on topical agents containing anthralin (a treatment associated to side effects such as skin staining and irritation), applied as lotions, creams, shampoos and ointments on the skin surface, locally, to treat psoriasis [33]. Similarly, topical use of apiin and hyperoside, after prolonged and repeated exposure to river waters or after their concentration (or extraction from plants) considering the ≤5 µg/L concentration could also work directly on the inflammatory region to improve the symptoms of psoriasis, avoiding adverse systemic effects thanks to the topical administration route [34]. Overall, the in silico findings described in this work, alongside the importance of protecting from pollution the riverine habitats [35,36,37,38,39] that are particularly rich in forests, living organisms in turn endowed with enormous benefits for human life [40,41,42,43,44,45], all concur to highlight the role that pristine rivers have on health and wellbeing of individuals.

## 4. Conclusions

Clean river water is rich in plant metabolites, which are endowed with beneficial therapeutic properties. For example, we demonstrated by an in silico approach that apiin and hyperoside, secondary plant metabolites present in river waters, could have inhibitory potential towards NF-kB, IL-17 and IL-36, the protein targets typical of psoriasis, resulting in binding affinities which are even higher than inhibitors found in the literature. Their therapeutic potential is corroborated by the experimentally-found antipsoriatic activity of their hydrolysis products, apigenin and quercetin (in turn a river plant metabolite), respectively. The low skin permeation (logKp < −8) we predicted for apiin and hyperoside led us to hypothesize their possible utilization as topical antipsoriatic therapeutics. In particular, the PAINS (pan-assay interference compounds) property prediction excluded any tendency for apiin to interact nonspecifically with the numerous targets involved in the biological cellular pathways, indicating this phytochemical as an interesting lead compound in psoriasis therapy. Overall, the findings of this study point to the potential antipsoriatic effect of river plant metabolites which could result from both multiple and prolonged river water applications on the skin (or river bathing) and concentration/isolation of metabolites from plant sources. Of course, there are many other medicinal compounds present within river waters as well as being emitted by riparian plant life, which have the potential to be used as treatments for a wide variety of ailments. These are also worth further investigation. However, the results found in the present study already emphasize the importance of protecting rivers, and in particular, preserving river water quality, biodiversity and the structure of riparian vegetation in order to allow for an increase in river frequentation for both recreational and therapeutic purposes. Perhaps giving legal rights to rivers would help to both end their abuse and remind us of the many benefits they provide. Then, like people have done in the Ganges for millennia, we will return to the waters for their holistic health properties as well as for a refreshing and relaxing dip.

## Supplementary Materials

The following supporting information can be downloaded at: https://www.mdpi.com/article/10.3390/ijerph19052502/s1, Tables S1 and S2: Predicted chemico-physical and pharmacokinetic properties for Apiin and Hyperoside as computed by SwissADME are displayed.
