# Supplementary material for: The Healing Power of Clean Rivers: In Silico Evaluation of the Antipsoriatic Potential of Apiin and Hyperoside Plant Metabolites Contained in River Waters
Source: Int J Environ Res Public Health. 2022 Feb 22;19(5):2502. doi: 10.3390/ijerph19052502 (PMC8909116; doi:10.3390/ijerph19052502)
Supplement: Supplementary file 1 [file ijerph-19-02502-s001.zip › ijerph-1541291-supplementary.pdf]

Supporting Information

The healing power of clean rivers: in silico evaluation of the antipsoriatic potential of apiin and hyperoside plant metabolites contained in river waters

Valentina Roviello¶[1], Melinda Gilhen-Baker¶[2], Caterina Vicidomini[3], and Giovanni N. Roviello[3,*]

[1] Department of Chemical, Materials and Industrial Production Engineering (DICMaPI), University of Naples Federico II, Piazzale V. Tecchio 80, 80125 Naples, Italy
[2] Faculty of Physical Medicine and Rehabilitation, Georgian State Teaching University of Physical Education and Sport, 49, Chavchavadze avenue, 0162 Tbilisi, Georgia
[3] Istituto di Biostrutture e Bioimmagini IBB - CNR Mezzocannone Site and Headquarters; I-80145 Naples, Italy

* Correspondence: giroviel@unina.it; Tel.: +39 0812534585
¶These authors contributed equally to this work.

Table S1 Predicted chemico-physical and pharmacokinetic properties for Apiin computed by SwissADME (http://www.swissadme.ch/index.php accessed on 8th November 2021)

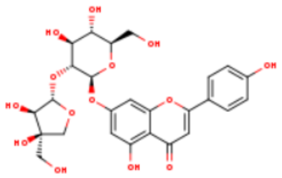

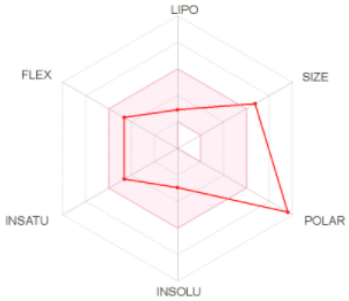

SMILES	OC[C@H]1O[C@@H](Oc2cc(O)c3c(c2)oc(cc3=O)c2ccc(cc2)O)[C@@H]([C@H]([C@@H]1O)O)O[C@@H]1OC[C@]([C@H]1O)(O)CO
**Physicochemical Properties**	
Formula	C26H28O14
Molecular weight	564.49 g/mol
Num. heavy atoms	40
Num. arom. heavy atoms	16
Fraction Csp3	0.42
Num. rotatable bonds	7
Num. H-bond acceptors	14
Num. H-bond donors	8
Molar Refractivity	132.56
TPSA	228.97 Å²
**Lipophilicity**	
Log $P_{o/w}$ (iLOGP)	2.31
Log $P_{o/w}$ (XLOGP3)	-0.36
Log $P_{o/w}$ (WLOGP)	-1.49
Log $P_{o/w}$ (MLOGP)	-3.16
Log $P_{o/w}$ (SILICOS-IT)	-0.72
Consensus Log $P_{o/w}$	-0.68

**Water Solubility**	
Log *S* (ESOL)	-2.95
Solubility	6.38e-01 mg/ml ; 1.13e-03 mol/l
Class	Soluble
Log *S* (Ali)	-3.99
Solubility	5.83e-02 mg/ml ; 1.03e-04 mol/l
Class	Soluble
Log *S* (SILICOS-IT)	-1.92
Solubility	6.83e+00 mg/ml ; 1.21e-02 mol/l
Class	Soluble
**Pharmacokinetics**	
GI absorption	Low
BBB permeant	No
P-gp substrate	Yes
CYP1A2 inhibitor	No
CYP2C19 inhibitor	No
CYP2C9 inhibitor	No
CYP2D6 inhibitor	No
CYP3A4 inhibitor	No
Log K_p (skin permeation)	-10.00 cm/s
**Druglikeness**	
Lipinski	No; 3 violations: MW>500, NorO>10, NHorOH>5
Ghose	No; 3 violations: MW>480, WLOGP<-0.4, MR>130
Veber	No; 1 violation: TPSA>140
Egan	No; 1 violation: TPSA>131.6
Muegge	No; 3 violations: TPSA>150, H-acc>10, H-don>5
Bioavailability Score	0.17
**Medicinal Chemistry**	
PAINS	0 alert
Brenk	0 alert
Leadlikeness	No; 1 violation: MW>350
Synthetic accessibility	6.08

Table S2 Predicted chemico-physical and pharmacokinetic properties for Hyperoside computed by SwissADME (http://www.swissadme.ch/index.php accessed on 8th November 2021)

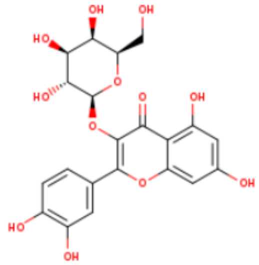

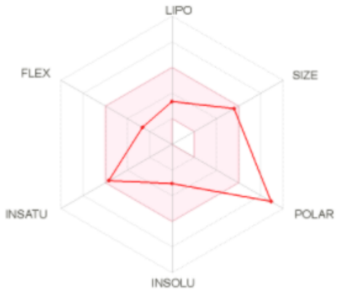

SMILES OC[C@H]1O[C@@H](Oc2c(oc3c(c2=O)c(O)cc(c3)O)c2ccc(c(c2)O)O)[C@@H]([C@H]([C@H]1O)O)O

Physicochemical Properties	
Formula	C21H20O12
Molecular weight	464.38 g/mol
Num. heavy atoms	33
Num. arom. heavy atoms	16
Fraction Csp3	0.29
Num. rotatable bonds	4
Num. H-bond acceptors	12
Num. H-bond donors	8
Molar Refractivity	110.16
TPSA	210.51 Å²

Lipophilicity	
Log $P_{o/w}$ (iLOGP)	1.45
Log $P_{o/w}$ (XLOGP3)	0.36
Log $P_{o/w}$ (WLOGP)	-0.54
Log $P_{o/w}$ (MLOGP)	-2.59
Log $P_{o/w}$ (SILICOS-IT)	-0.59
Consensus Log $P_{o/w}$	-0.38

Water Solubility	
Log *S* (ESOL)	-3.04
Solubility	4.23e-01 mg/ml ; 9.10e-04 mol/l
Class	Soluble
Log *S* (Ali)	-4.35
Solubility	2.10e-02 mg/ml ; 4.51e-05 mol/l
Class	Moderately soluble
Log *S* (SILICOS-IT)	-1.51
Solubility	1.43e+01 mg/ml ; 3.08e-02 mol/l
Class	Soluble

Pharmacokinetics	
GI absorption	Low
BBB permeant	No
P-gp substrate	No
CYP1A2 inhibitor	No
CYP2C19 inhibitor	No
CYP2C9 inhibitor	No
CYP2D6 inhibitor	No
CYP3A4 inhibitor	No
Log K_p (skin permeation)	-8.88 cm/s

Druglikeness	
Lipinski	No; 2 violations: NorO>10, NHorOH>5
Ghose	No; 1 violation: WLOGP<-0.4
Veber	No; 1 violation: TPSA>140
Egan	No; 1 violation: TPSA>131.6
Muegge	No; 3 violations: TPSA>150, H-acc>10, H-don>5
Bioavailability Score	0.17

Medicinal Chemistry	
PAINS	1 alert: catechol_A
Brenk	1 alert: catechol
Leadlikeness	No; 1 violation: MW>350
Synthetic accessibility	5.32
